# Utilization of Human Induced Pluripotent Stem Cells-Derived *In vitro* Models for the Future Study of Sex Differences in Alzheimer’s Disease

**DOI:** 10.3389/fnagi.2021.768948

**Published:** 2021-11-04

**Authors:** Sopak Supakul, Hideyuki Okano, Sumihiro Maeda

**Affiliations:** Department of Physiology, Keio University School of Medicine, Tokyo, Japan

**Keywords:** Alzheimer’s disease, sex difference, hormone therapy, iPSCs (induced pluripotent stem cells), *in vitro* model

## Abstract

Alzheimer’s disease (AD) is an aging-dependent neurodegenerative disease that impairs cognitive function. Although the main pathologies of AD are the aggregation of amyloid-beta (Aβ) and phosphorylated Tau protein, the mechanisms that lead to these pathologies and their effects are believed to be heterogeneous among patients. Many epidemiological studies have suggested that sex is involved in disease prevalence and progression. The reduction of sex hormones contributes to the pathogenesis of AD, especially in females, suggesting that the supplementation of sex hormones could be a therapeutic intervention for AD. However, interventional studies have revealed that hormone therapy is beneficial under limited conditions in certain populations with specific administration methods. Thus, this suggests the importance of identifying crucial factors that determine hormonal effects in patients with AD. Based on these factors, it is necessary to decide which patients will receive the intervention before starting it. However, the long observational period and many uncontrollable environmental factors in clinical trials made it difficult to identify such factors, except for the *APOE* ε4 allele. Induced pluripotent stem cells (iPSCs) derived from patients can differentiate into neurons and recapitulate some aspects of AD pathogenesis. This *in vitro* model allows us to control non-cell autonomous factors, including the amount of Aβ aggregates and sex hormones. Hence, iPSCs provide opportunities to investigate sex-dependent pathogenesis and predict a suitable population for clinical trials of hormone treatment.

## Introduction

Alzheimer’s disease (AD) is a neurodegenerative disease associated with cognitive decline over time and has the highest prevalence compared to other types of dementia worldwide (Mayeux and Stern, 2012; Alzheimer’s Association, 2021). Although several clinical trials targeting the main components of AD neuropathology, such as amyloid-beta (Aβ) peptide and phosphorylated Tau (pTau) protein have failed to achieve good outcomes (Mehta et al., 2017; Mullane and Williams, 2018; Yiannopoulou et al., 2019), it is believed that the heterogeneous nature of the disease mechanism among patients with AD determines the response to the drug (Devi and Scheltens, 2018). Even in the only successful clinical trial of aducanumab, the heterogeneity of disease progression is reported to be the reason that in one cohort, they did not show statistically significant drug effects (Knopman et al., 2021). Sex has been reported to affect the pathogenesis of neurodegenerative diseases, such as Parkinson’s disease (PD) (Wooten et al., 2004) and AD (Dubal, 2020). Females have a higher risk of developing AD than males, while males have a higher risk of developing PD. However, male patients with AD have a higher prevalence of mild cognitive impairment (MCI) and faster progression to AD, which is associated with a higher mortality rate than females (Barnes et al., 2003; Mielke, 2018; Strand et al., 2018; Davis et al., 2020; Dubal, 2020). A recent study suggested that higher expression of *KDM6A*, which is the escape of X chromosome inactivation, contributes to greater resilience to AD in females (Davis et al., 2020). However, this mechanism of cell-autonomous effects in AD is different from that in PD. The expression level of the *SRY* gene on the Y chromosome regulates the response to pathogens related to PD (Lee et al., 2019). These sex differences may make it difficult to interpret the negative results of clinical trials for AD.

In terms of non-cell-autonomous causes, since most patients with AD are often late-onset, when the level of sex steroid hormones is decreased in both males (testosterone) and females (estrogen and progesterone), sex steroid hormones are suggested to contribute to the pathogenesis of the disease. Therefore, hormone replacement therapy (HRT), which is often used in patients with decreased hormone levels, has also been suggested as a potential intervention for patients with AD. However, many previous randomized clinical trials (RCTs) on the effects of sex hormones on cognitive function and AD development showed varying results between cohorts/studies. In addition, the discrepancy in the results between clinical and preclinical trials was also an issue. In particular, because mice maintain hormone levels throughout their life span, mouse models of AD have some limitations in studying sex differences and hormonal effects on AD pathogenesis (Dubal et al., 2012). In addition, Aβ aggregates have been reported to induce apoptotic changes in human neurons but not in mouse neurons (Espuny-Camacho et al., 2017). These results indicate the need for developing a robust AD model of human cells capable of recapitulating both autonomous and non-autonomous effects, especially for the investigation of sex effects on AD. This perspective aimed to summarize the results and problems of previous clinical studies using sex hormones for AD patients and discuss the potential of next generation *in vitro* AD models, especially induced pluripotent stem cells (iPSCs), together with our preliminary results using iPSC-derived neurons.

## Past Clinical Trials Investigating Sex Steroid Hormone Therapy Effects on Alzheimer’s Disease

The neuropathology of AD usually starts developing over 10 − 20 years before the onset of symptoms and progresses with age. Therefore, it is suspected that aging factors, including the reduction of sex hormone levels, contribute to AD development. Many epidemiological studies have suggested that AD has a high prevalence in menopausal elderly females, whose levels of sex hormones, including estrogens and progesterone, have decreased compared with levels at reproductive age (Santoro and Sutton-Tyrrell, 2011). Likewise, biologically active testosterone decreases with age in males. This decrease may also contribute to AD development in males (Moffat, 2005; Lv et al., 2016). In AD mouse models that had undergone gonadectomy, it was also found that treatment with sex steroid hormones could slow the progression of Aβ accumulation, decrease Tau hyperphosphorylation, and improve working memory (Yue et al., 2005; Carroll et al., 2007). Many clinical trials have attempted to reveal the beneficial effects of sex hormones on AD, mainly estrogen and progesterone, in menopausal females with AD. However, clinical studies have shown mixed results, and the association between sex hormones and AD remains elusive.

### Observational Studies of Hormone Replacement Therapy in Alzheimer’s Disease

Early observational studies showed a compelling beneficial effect of estrogen on AD in cognitive tests and positron emission tomography scans using bolus injected [^15^O] water. Honjo et al. (1989) treated female patients with AD with oral conjugated estrogen (estrone sulfate as the main component) and suggested an improvement in cognitive function after 6-weeks of treatment (Honjo et al., 1989). Henderson et al. (1996) compared female patients with AD who received estrogen replacement therapy with female and male patients with AD who did not receive estrogen replacement therapy. They showed that female patients with AD who received estrogen showed significantly better performance on cognitive function tests than female and male patients who did not receive estrogen (Henderson et al., 1996). In addition, Resnick et al. (1997) showed that female patients who received HRT had better cognitive function tests, especially those related to visual memory (Resnick et al., 1997). A subsequent study also showed that HRT users had better regional cerebral blood flow (rCBF) in the right parahippocampal gyrus, right precuneus, and right frontal regions during memory tasks, suggesting better activation of the brain areas that are severely affected in patients with AD (Resnick et al., 1998).

### Randomized Clinical Trials of Hormone Replacement Therapy in Alzheimer’s Disease

Based on the positive results of the observational studies, RCTs were launched. However, the RCTs showed various results, including positive, partially positive, and negative effects of HRT (Table 1). Some of the first RCTs indicated that HRT had no effect on AD or adversely increased the risk of developing AD, contrary to expectations. Henderson et al. (2000) showed that the treatment of conjugated equine estrogens (CEE) for 4 weeks in postmenopausal female patients with mild to moderate AD did not result in significant improvement in cognitive function compared to the placebo group (Henderson et al., 2000). Mulnard et al. (2000) treated menopausal female patients with AD with high and low doses of CEE for 1 year, a longer period than previous studies, and found that CEE did not affect attenuating AD (Mulnard et al., 2000). One large clinical cohort, the Women’s Health Initiative Memory Study that included more than 4,000 menopausal female patients who were receiving CEE and/or medroxyprogesterone acetate for more than 4 years, also revealed negative or even adverse effects of hormone treatment. Treatment with estrogen and progesterone has been reported to have adverse effects that may promote the development of AD. Thus, the opposed estrogen therapy, which is a form of estrogen therapy supplemented with progesterone to prevent the adverse effects of excessive estrogen alone, such as enhancing the risk of endometrial cancer development, was less beneficial in preventing the development of AD (Shumaker et al., 2003; Craig et al., 2005).

**TABLE 1:** Summary of selected randomized clinical trials (RCTs) of the hormone replacement therapy (HRT) effects on cognitive function among postmenopausal women of AD or non-AD group.

No.	Comparisons	Targets	N	Age range	Route of administration	Regimen/Dose	Duration	Outcome measures	Main results	HRT effectiveness	References
1	Transdermal 17β-E2 **VS** Placebo	Postmenop ausal women with mild to moderate AD	12	66–89 y.o.	Transdermal	17β-E2 0.05 mg/day	8 weeks	**Primary:** SRT, Paragraph Recall, VR, SCWT, TMT, Verbal fluency, Token Test; **Secondary:** Dementia and psychiatric status: MMSE, BMIC, BPRS; Other laboratory measures: Serum E2, E1, FSH assays, IGF-1, IGFBP-3 assays, Serum epinephrine, and nor-epinephrine assays	(1) 17β-E2 treatment improved attention and verbal memory (2) Plasma level of estradiol was positively correlated with enhanced verbal memory	Yes (attention and verbal memory)	Asthana et al., 1999
2	CEE **VS** Placebo	Postmenop ausal women with mild to moderate AD	36	CEE [77 ± 1.4]; Placebo [78 ± 1.0]	Oral	CEE 1.25 mg/day	16 weeks	**Primary:** ADAS-Cog; **Secondary:** clinician- rated GIC, caregiver-rated functional status	(1) No significant differences in cognitive function tests between treatment groups	No	Henderson et al., 2000
3	High dose or low dose CEE **VS** Placebo	Postmenop ausal women with mild to moderate AD who had previous hysterectomy history	97	56–91 y.o.	Oral	High dose CEE 1.25 mg/day, Low dose CEE 0.625 mg/day	12 months	**Primary:** CGIC scale; **Secondary:** MMSE, CDR, Ham-D, MAAACL-R, ADAS-cog, Emotional Face Recognition Test, New Dot Test, Letter Cancellation, TMT-A, Digit Symbol, Category Fluency, Letter Fluency, Grooved Pegboard Test, Finger Tapping Test, BDRS, the dependency scale	(1) ERT for 1 year did not improve CGIC scale in women with mild to moderate AD compared to placebo group (2) Women randomized to ERT showed a worsening CDR scale	No	Mulnard et al., 2000
4	CEE or CEE + MP4 progesterone **VS** Tacrine	Women with mild to moderate AD	55	53–85 y.o.	Oral	CEE 0.625 mg/day; MP4 100 mg/day; Tacrine 40-160 mg/day	6 months	**Primary:** MMSE, HVLT, BNT, COWAT, GDS, HDS, IADL	(1) Efficacy for cognition and mood of estrogen + progesterone was similar to tacrine	Yes	Yoon et al., 2003
5	CEE + MPA **VS** CEE + Placebo	Postmenop ausal women without probable dementia	4532	≥ 65 y.o.	Oral	CEE 0.625 mg/day, MPA 2.5 mg/day	4.05 years	**Primary:** Incidence of probable dementia; **Secondary:** Incidence of mild cognitive impairment	(1) Estrogen + progestin therapy increased the risk for probable dementia in postmenop ausal women aged 65 years or older (2) Estrogen + progestin did not prevent MCI in postmenop ausal women aged 65 years or older	No	Shumaker et al., 2003
6	17β-E2 **VS** Placebo	Healthy menopausal women	17	57 y.o. (SD 6.89)	Transdermal	Patches applied 2 times/week (Active estrogen had a release rate of 0.1 mg E2/day)	10 weeks	**Primary:** Stroop Task, WAIS-R, Grooved Pegboard Test, CVLT, WMS-R, WMS-R Visual Reproduction, ROCF, WCST, COWAT, TMT, WAIS-R Picture Arrangement subtest raw score	(1) Recent menopausal women showed more positive changes to neuropsychiatric measures of executive functioning	Yes (executive functioning)	Dunkin et al., 2005
7	SERM (Raloxifene) **VS** Placebo	Menopausal women with osteoporosis	5386	35.7–80.9 y.o.	Oral	Raloxifene 60 mg/day, Raloxifene 120 mg/day	3 years	**Primary:** The effect of raloxifene on vertebral fractures; **Secondary:** Development of mild cognitive impairment and dementia	(1) Women taking high dose (120 mg/day) of raloxifene has lower risk of MCI	Yes	Yaffe et al., 2005
8	E2 + Norethisterone **VS** Placebo	Menopausal women with probable AD	55	65–89 y.o.	Oral	E2 1 mg/day + Norethisterone 0.5 mg/day	12 months	**Primary:** DRS, CERAD, GDS, Barthel Index	(1) Women without *APOE* ε4 allele treated with E2 showed a greater reduction in depression screening score compared to women with *APOE* ε4 allele (2) Women without *APOE* ε4 allele treated with E2 showed better mood, word learning memory score, and GDS score	Partially (according to *APOE* allele)	Valen-Sendstad et al., 2010
9	Continued HT **VS** Discontinued HT	Cognitively normal postmenop ausal women at risk for AD and receiving estrogen HT	53	50–65 y.o.	Oral/Transdermal	Following each original HT regimen	2 years	**Primary:** Cognitive status: ACT, BVRT, BNT, Color Trail Making Test, DKEFS, RCFT, WAIS-III, WMS, MFQ. Regional cerebral metabolism: FDG-PET	(1) Women who continued HT showed less decline of the posterior cortical metabolism and preserved anterior cortical metabolism (2) Women who took 17β-E2 performed better in verbal memory compared to CEE (3) Women taking both estrogen and progesterone showed lower metabolism of mesial and inferior lateral temporal regions and the inferior frontal cortex, contralateral to Broca’s area (4) Women with more years of endogenous estrogen exposure showed a preserved metabolism same as women taking unopposed estrogen	Partially (women took transdermal 17β- E2, unopposed E2, longer endogenous E2 exposure)	Silverman et al., 2011
10	High or Low dose of E2 ± progestin **VS** Placebo	Postmenop ausal women with mild-moderate AD	43	55–85 y.o.	Transdermal (17β-E2)/Oral (MPA, placebo)	1) Low dose unopposed HT: 50μg transdermal 17β-E2 and a placebo tablet daily, 2) Low dose opposed HT: 50μg transdermal 17β-estradiol and 2.5 mg of MPA daily, 3) High dose unopposed HT: 100μg transdermal 17β-E2 and a placebo tablet, 4) High dose opposed HT: 100μg transdermal 17β-E2 and 2.5 mg of MPA daily, or 5) Placebo skin patch and placebo tablet daily	12 months	**Primary:** BNT, Figural Memory Test, CFT, Visual Paired Associates, Paragraph Recall, List Learning, TMT-B, SCWT, POMS, and laboratory tests	(1) 3 months administration of transdermal 17β-E2 showed positive effects on semantic memory and visual memory (2) Within 3 months of treatment, opposed 17 β-estradiol administration showed greater benefits for visual memory compared to unopposed therapy	Yes	Wharton et al., 2011
11	Continued HT **VS** Discontinued HT	Postmenop ausal women with AD risk	45	50–65 y.o.	Oral	Following each original HT regimen: 18 women using CEE 0.625 mg (9 monotherapy, 9 concurrent with progestin) and 36 women using 17β-E2 0.1mg (12 monotherapy, 24 concurrent with progestin)	2 years	**Primary:** FDG-PET of regional brain metabolism	(1) Metabolic decline in the medial frontal cortex found in women who discontinued HT (2) Metabolic decline in the posterior/precuneus area found in women who discontinued 17β-E2, continued CEE, and continued opposed HT (either 17β-E2 or CEE)	Partially (women took unopposed transdermal 17β-E2)	Rasgon et al., 2014
12	SERM (Raloxifene) **VS** Placebo	Postmenop ausal women with mild to moderate late onset AD	39	68–84 y.o.	Oral	Raloxifene 120 mg/day	12 months	**Primary:** ADAS-cog	(1) ADAS-cog scores and other secondary outcome measures were not differ between treatment groups	No	Henderson et al., 2015
13	Oral CEE **VS** Transdermal 17β-E2 **VS** Placebo pills and patch	Healthy women who were within 5 to 36 months past menopause	68	52–65 y.o.	Oral/Transdermal	CEE 0.45 mg/day, Transdermal 17β-E2 50 μg/day	4 years	**Primary:** PiB-PET of Aβ deposition	(1) Women took transdermal 17β-E2 had lower Aβ deposition compared to placebo (2) Women with APOE ε4 allele and treated with 17β-E2 had lower Aβ deposition compared to placebo and oral CEE (3) Among non- carriers of APOE ε4 allele, HT did not cause changes in PiB PET imaging	Partially (women treated with transdermal 17β-E2 and had APOE ε4 allele)	Kantarci et al., 2016
14	Percutaneous E2 gel + Oral MP4 **VS** Placebo	Postmenopausal women with MCI	35	57–82 y.o.	Transdermal/Oral	E2 gel 2 mg/day, MP4 100 mg/day	24 months	**Primary:** ADAS-cog, K-MMSE, K-MoCA	(1) Progression rate to dementia was higher in placebo group compared to HT group (2) HT group showed a reduced deterioration of K-MoCA score compared to placebo	Yes	Yoon et al., 2018

*ACT: Auditory Consonant Trigrams; AD: Alzheimer’s Disease; ADAS-Cog: Alzheimer’s Disease Assessment Scale-Cognitive subscale; ADCS-CGIC: Alzheimer’s Disease Cooperative Study Clinical Global Impression of Change; BDRS: Blessed Dementia Rating Scale; BIMC: Blessed Memory Information and Concentration Test; BNT: Boston Naming Test; BPRS: Brief Psychiatric Rating Scale; BVRT: Benton Visual Retention Test; CDR: Clinical Dementia Rating Scale; CEE: Conjugated equine estrogens; CERAD: Consortium to Establish a Registry for Alzheimer’s Disease; CGIC: Clinical Global Impression of Change; COWAT: Controlled Oral Word Association Test; CVLT: California Verbal Learning Test; DKEFS: Delis Kaplan Executive Function System; DRS: Dementia Rating Scale; ERT: Estrogen Replacement Therapy; E1: Estrone; E2: Estradiol; FDG-PET: Fluorodeoxyglucose-Positron Emission Tomography; FSH: Follicle-Stimulating Hormone; GDS: Global Deterioration Scale; GDS: Geriatric Depression Scale; GIC: Global Impression of Change; Ham-D: Hamilton Depression Rating Scale; HDS: Hamilton Depression Scale; HT: Hormone Therapy; HVLT: Hopkins Verbal Learning Test; IADL: Instrumental Activities of Daily Living; IGF-1: Insulin-like Growth Factor-1; IGFBP-3: Insulin-like Growth Factor Binding Protein-3; K-MMSE: the Korean version of Mini-Mental State Examination; K-MoCA: the Korean version of Montreal Cognitive Assessment; MAAACL-R: Multiple Affect Adjective Checklist–Revised; MCI: Mild Cognitive Impairment; MFQ: Memory Function Questionnaire; MMSE: Mini-Mental State Examination; MPA: Medroxyprogesterone acetate; MP4: Micronized progesterone; PiB-PET: Pittsburgh compound B- Positron Emission Tomography; P4: Progesterone; RCFT: Rey-Osterrieth Complex Figure Test; ROCF: Rey-Osterreith Complex Figure; SCWT: Stroop Color-Word Interference Test; SERM: Selective Estrogen Receptor Modulator; SRT: Buschke Selective Reminding Test; TMT: Trail Making Test; TMT-A: Trail Making Test-Part A; TMT-B: Trail Making Test-Part B; VR: Visual Reproduction Test; WAIS-III: Wechsler Adult Intelligence Scale-3rd Edition; WMS-III: Wechsler Memory Scale-3rd Edition; WAIS-R: Wechsler Adult Intelligence Scale-Revised; WMS-R: Wechsler Memory Scale-Revised; 17β-E2: 17β-estradiol.*

Due to the negative results of RCTs during the early 2000s, the following RCTs focused more on the stratification of patient characteristics (e.g., age, cognitive status, *APOE* genotype, duration after menopause), along with the formulation of drugs and the route of administration (e.g., oral CEE, transdermal 17β-estradiol). These RCTs were finally able to assert the beneficial effects of estrogen in human clinical trials. Valen-Sendstad et al. (2010) showed that females without the *APOE* ε4 allele (a major genetic risk for AD) received estradiol and norethisterone, they had better mood and depression than females with the *APOE* ε4 allele (Valen-Sendstad et al., 2010). Silverman et al. (2011) compared the continued and discontinued HRT effects among cognitively normal postmenopausal females with a family history of AD and showed the following results: (i) 17β-estradiol had a better effect than CEE; (ii) unopposed estrogen therapy preserved more cortical metabolism compared to the opposed one; and (iii) females with longer endogenous estrogen exposure showed relatively preserved metabolism of specific brain areas more than females with shorter endogenous estrogen exposure (Silverman et al., 2011). Moreover, the Kronos Early Estrogen Prevention study, which included only recent postmenopausal females, concluded that 17β-estradiol could lower Aβ deposition, especially in those with *APOE* ε4 among the recent menopausal female population (Kantarci et al., 2016). In addition to conventional transdermal 17β-estradiol or oral CEE, selective estrogen receptor modulators as another form of medication are also being investigated for their effects on improving cognitive function. A study by Wang et al. (2020) showed that PhytoSERM [an estrogen receptor beta (ERβ) modulator comprised of genistein, daidzein, and S-equol] improved cognitive function, especially for verbal learning and executive function (Wang et al., 2020).

Similar to estrogen and progesterone, testosterone levels also decrease with age, and low testosterone levels contribute to cognitive decline in elderly males (Moffat, 2005; Lv et al., 2016). Although only a few RCTs have evaluated the effect of testosterone on cognitive function, studies of short-term testosterone replacement therapy in elderly patients with MCI or AD found improvement in cognitive function after treatment, especially for spatial and verbal memory (Tan and Pu, 2003; Cherrier et al., 2005), while long-term therapy of testosterone showed only modest or no significant improvement (Asih et al., 2015; Huang et al., 2016; Wahjoepramono et al., 2016). Meanwhile, Huang et al. (2015) reported no significant cognitive improvement after testosterone administration, even among females with low testosterone levels (Huang et al., 2015).

## Next Generation *in vitro* Models of AD and Future Utilization for the Study of Sex Difference

### Alzheimer’s Disease Modeling Using Induced Pluripotent Stem Cells

After the establishment of iPSCs technology by the group of Shinya Yamanaka (Takahashi and Yamanaka, 2006; Takahashi et al., 2007), human cell models of many diseases, including neurological disorders, have been generated using iPSCs (Okano and Yamanaka, 2014; reviewed in Imaizumi and Okano, 2014; Rowe and Daley, 2019; Sharma et al., 2020). For AD, iPSCs were first established from fibroblasts obtained from patients with familial Alzheimer’s disease (fAD) with mutations in *PS1* (A246E) and *PS2* (N141l) (Yagi et al., 2011), and the iPSCs were differentiated into neurons. These neurons induced by iPSCs recapitulated the phenotypes of AD, such as increased Aβ_42_ levels and responded to AD targeting drugs, such as γ-secretase inhibitors and modulators. The following models of fAD with different mutations also showed similar phenotypes (reviewed in Zhang et al., 2016). In addition to the models of familial AD cases, a sporadic AD model of iPSC-derived neurons was established in 2012, in which increased Aβ levels, pTau protein, and other AD phenotypes were observed (Israel et al., 2012). Recent studies using iPSCs focused on sAD risk genes that increase the risk of developing AD, such as *SORL1* (Young et al., 2015) and *APOE*. However, since the comparison of iPSCs from mutation carrier and non-carrier cannot exclude the effects of other SNPs that are different between the mutation carrier and non-carrier, using genome-editing technology like CRISPR/Cas9 allows us to introduce only the mutation of interest and examine the effects. Knupp et al. (2020) generated the *SORL1* knockout iPSCs using CRISPR/Cas9 and could conclude that the loss of *SORL1* induced early endosome enlargement (Knupp et al., 2020). Furthermore, another study utilized CRISPR/Cas9 to establish *APOE* ε3/ε3 and *APOE* ε4/ε4 hiPSC lines that have equal genomic background except for the *APOE* gene. Then, they investigated functions of *APOE* genotypes on diverse cell types including neurons, astrocytes, and microglia-like cells (Lin et al., 2018). Since the AD models established from iPSCs can reflect the phenotypes of the disease and respond to drugs, this tool can be used to investigate both cell-autonomous and non-cell-autonomous effects arising from sex differences within the AD population.

### Induced Pluripotent Stem Cell-Derived Neuronal Models Recapitulated Alzheimer’s Disease Pathology and Responded to Estradiol Treatment

**FIGURE 1:**
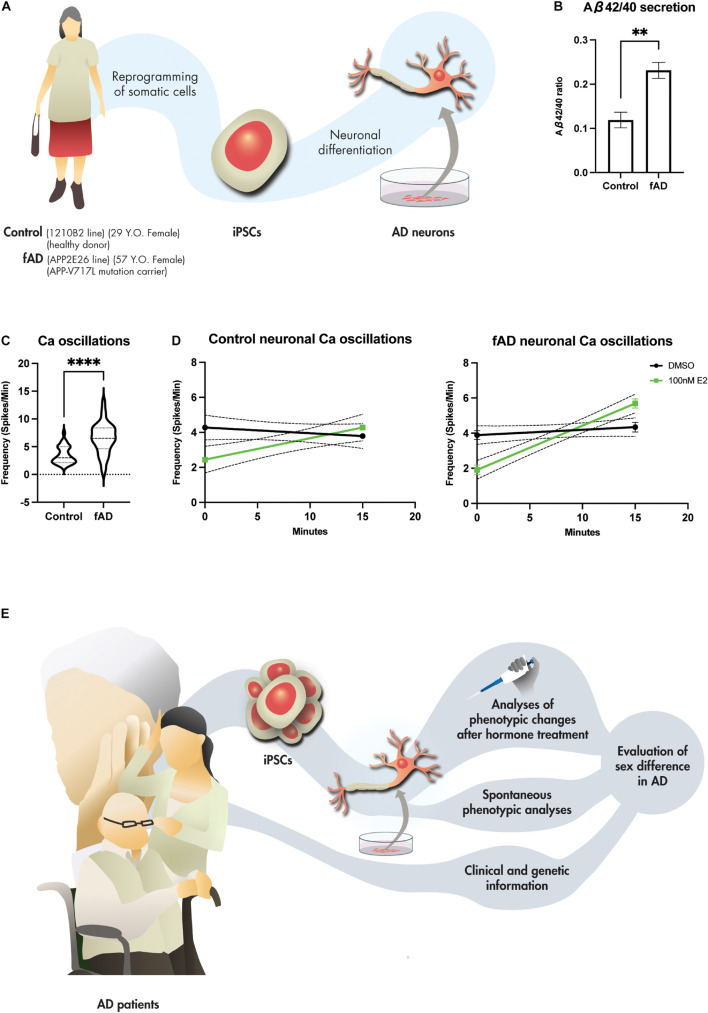
iPSC-derived neurons for the future study of sex difference in AD. **(A)** Generation of iPSC-derived neurons from female healthy (1210B2 line) and fAD (APP2E26 line) donors. **(B)** Aβ_42/40_ ratio of the iPSC-derived neurons of healthy (*n* = 4) and fAD (*n* = 4) donors measured at 45 div. Bars, mean ± SEM. **p < 0.01 (unpaired *t*-test). **(C)** Neuronal hyperactivation seen in fAD-derived neurons measured by Ca imaging using Fluo-8 indicator after 45 div (*n* = 32 versus 32). ****p < 0.001 (Mann Whitney test). **(D)** 17β-estradiol (E2) responses of iPSC-derived neurons measured by Ca imaging using Fluo-8 indicator at 45 div of 1210B2 line and APP2E26 line before (0 min) and after (15 min) treatment with 100 nM E2 (*n* = 45 versus 45). *p* = 0.3331 (1210B2, DMSO); *p* = 0.0010 (1210B2, 100 nM E2); *p* = 0.2289 (APP2E26, DMSO); *p* < 0.0001 (APP2E26, 100 nM E2) (simple linear regression). **(E)** Stratification and investigation of the hormone therapy effects in AD utilizing iPSC-derived neuronal models.

In our analyses of the neurons differentiated from human iPSCs of healthy female donor (1210B2 line) and female donor of fAD (*APP* V717L line) (Figure 1A) (Kondo et al., 2013; Nakagawa et al., 2014), secreted Aβ peptides were measured by enzyme-linked immunosorbent assay. Neurons from fAD donor increased Aβ_42__/__40_, indicating that iPSC-derived neurons recapitulated the disease pathophysiology of AD (Figure 1B) (Jack et al., 2013; Lee et al., 2020). Furthermore, iPSC-derived neurons of the fAD donor increased the frequency of Ca imaging using Fluo-8 compared to healthy control (Figure 1C). This finding was reminiscent of the finding of Zott et al. (2019) that neuronal hyperactivation in AD mouse models is caused by Aβ-induced accumulation of perisynaptic glutamate (Zott et al., 2019). These neurons derived from iPSCs also responded to short-time exposure to 17β-estradiol. Exposure to 17β-estradiol for 15 min increased the Ca oscillation (Figure 1D). Previously, Zhang et al. (2010) also performed Ca imaging on human and mouse embryonic stem cell (ESC)-derived neurons after treatment with 17β-estradiol and showed increased neuronal firing (Zhang et al., 2010). Increased activation of neurons after acute treatment with estradiol is suggested to be mediated by the stimulation of L-type Ca^2+^ channels, which activate the MAPK/ERK pathway and promote the firing of neurons as part of the rapid signaling cascade (Sheldahl et al., 2008; Vega-Vela et al., 2017; Albert-Gascó et al., 2020). Shum et al. (2015) also treated iPSC-derived neurons from healthy donors with 17β-estradiol for 24 h and showed increased dendritic branching (Shum et al., 2015), suggesting promotion of neuronal microstructure by estrogen in the iPSC-derived models. The increased neuronal branching may explain the preserved cognitive function by HRT, as shown in previous RCTs. Further experiments such as the treatment of 17β-estradiol before/after Aβ treatment in iPSC-derived neurons will tell us whether 17β-estradiol protects neurons against oxidative stressor. Another study differentiated neurons from the iPSCs of the autism individual revealed an increased expression of androgen receptor and brain-derived neurotrophic factor after testosterone treatment, suggesting that the iPSC-derived neurons also respond to testosterone treatment (Adhya et al., 2018). These evidence suggest that the *in vitro* model using iPSCs responds to sex steroid hormone and has potential for future study of sex differences, including the effects of hormone therapy, among AD patients.

### Advantages of Induced Pluripotent Stem Cell-Derived *In vitro* Models for the Study of Neurodegenerative Diseases

Induced pluripotent stem cells (iPSCs) can hold the genetic information of the donors, thus allowing *in vitro* study of diseases with the donor’s genetic background (Stadtfeld and Hochedlinger, 2010). Non-cell-autonomous factors, such as the exposure time to hormones and the amount of Aβ aggregates, can also be controlled. Moreover, the iPSC-derived disease model can recapitulate the phenotypes of individual patients and can be used for disease stratification and drug screening of incurable neurodegenerative diseases. Previously, stratification and drug screening for sporadic disease were performed in amyotrophic lateral sclerosis (ALS). Motor neurons differentiated from patients with ALS-derived iPSCs showed multiple phenotypes, such as neurite retraction, lactate dehydrogenase leakage, increased cleaved caspase-3, and abnormal protein aggregation. These phenotypes *in vitro* matched the clinical manifestations and could stratify the disease (Fujimori et al., 2018). The study further identified ropinirole, a well-known medication for PD, as a drug candidate that could be used to treat patients with ALS. This *in vitro* study finally led to a successful clinical trial of ropinirole in patients with ALS (Morimoto et al., 2019; Okano et al., 2020; Keio University School of Medicine, 2021). The success of ALS indicated the possibility of stratification and prediction of drug response for patients with AD using iPSC models derived from patients along with their clinical information. Additionally, one recent study also generated the human iPSCs-derived model of microglia and confirmed that female sex and *APOE* genotype drive the microglial transcription profiles, thus indicating another important aspect of sex difference and *APOE* genotype in AD (Moser et al., 2021).

The cell-autonomous effect of different sexes on cell models can be observed through several readouts under pathogen-free conditions, while non-cell-autonomous phenotypic changes can be observed by exposing the cells to pathogens or drugs. Furthermore, genetic information (especially AD risk genes) and clinical information of the patients can be included in the stratification of the disease. The effects of hormone therapy in patients can be predicted by phenotypic changes based on the results of the iPSC model. Finally, stratification for hormone treatment using iPSCs from individual participants would yield a robust simulation before the clinical trial, although the cost and time for the generation of iPSCs and subsequent analysis of cell models from individual participants will be a barrier (Figure 1E). Currently, the development of the 3D model such as brain organoids has advanced the disease modeling using iPSCs and were able to recapitulate the patient’s pathophysiology (Chiaradia and Lancaster, 2020). For example, the 3D brain organoid could recapitulate the amyloid aggregation which 2D model could not (Gonzalez et al., 2018). In 2D culture, the secreted Aβ is released into the medium and cannot be concentrated high enough to aggregate. However, in 3D culture, the secreted Aβ can be constrained in the extracellular space of the brain organoids and can increase the concentration high enough to aggregate. Furthermore, the utilization of 3D organoid in combination with the co-culture with other CNS cells/structure like microglia and blood vessels would yield a better modeling of disease and can be used for observation of the hormone therapy in the future.

Nevertheless, the iPSC technology has some limitations such as the genetic alterations that could occur during the reprogramming of the somatic cells to the iPSCs. Even though new reprogramming methods using the non-integrating tools like Sendai virus and episomal vectors caused less damages than the first generation of the reprogramming with lentivirus (Kang et al., 2015), the genetic alterations can still occur. Thus, many of the tests such as karyotyping, genotyping, stem cell markers’ expressions are usually required to confirm the quality of the established iPSCs before using for the modeling of diseases. In addition to neurons differentiated from iPSCs, human neurons can also be induced directly from somatic cells, such as fibroblasts (Victor et al., 2018). Directly induced neurons (iNs) obtained from the fibroblasts preserved the aging status of the donors, while the aging status of the donor was erased in iPSCs. The iNs of sporadic AD patients could also reflect AD phenotypes, such as uncomplicated dendrites, reduced synapses, epigenetic erosion, and increased DNA damage (Mertens et al., 2021). Thus, AD models of iN cells can also be used to evaluate aging-dependent sex effects and could provide insights into sex and age-related changes in AD.

## Conclusion

At the human level, early observational studies of estrogen and progesterone replacement therapy have suggested that sex hormones are beneficial to postmenopausal female patients with AD. However, some of the RCTs showed negative results or adverse effects. Factors such as patient characteristics (e.g., age, *APOE* genotype, duration after menopause), formulation of therapeutic hormones, and route of administration were suggested to be associated with the positive/partially positive/negative outcomes of each study. These results suggest that hormone therapy could only be beneficial in certain populations (Maki, 2013). More recent RCTs showed positive effects of HRT in specific groups of AD patients after stratification of participants by age, duration of exposure to endogenous estrogen, *APOE* genotype, and formula of estrogen. Therefore, the stratification of the patients and the adjustment of the regimen before starting the trial are crucial steps to predict the precise outcomes of hormonal therapy on cognitive function.

In this regard, *in vitro* models, such as iPSCs that can recapitulate human disease development of individual donors, is a promising tool for studies that aim to stratify diseases with heterogeneity, including AD. Since the unknown factors that affect the outcomes of hormone treatment may still exist, the uncontrollable environmental factors in clinical trials may mask the beneficial effects of drugs. The iPSC-derived models can foster the discovery of such factors and redesign clinical trials. Although clinical studies at the human level need to be conducted, human cell models can provide significant evidence before proceeding to clinical trials. Our phenotypic analyses of human iPSC-derived neuronal models from AD and non-AD donors showed that the models could recapitulate AD pathology and respond to 17β-estradiol treatment. Hence, this platform of *in vitro* models using iPSCs provides opportunities to search for new potential factors that are crucial for the stratification of AD. This will allow us to search for a new treatment strategy in the future.

## Data Availability Statement

The raw data supporting the conclusions of this article will be made available by the authors, without undue reservation.

## Ethics Statement

The studies involving human participants were reviewed and approved by Keio University. The patients/participants provided their written informed consent to participate in this study.

## Author Contributions

SS involved in the study design, performing experiments, data analyses, and drafting the original manuscript. SM supervised the study design, experiments, data analyses, and revised the manuscript. HO involved in the critical revision of the manuscript. All authors contributed to the article and approved the submitted version.

## Conflict of Interest

HO is a founder scientist and a Scientific Advisory Board Member for SanBio Co., Ltd. and K Pharma Inc. The remaining authors declare that the research was conducted in the absence of any commercial or financial relationships that could be construed as a potential conflict of interest.

## Publisher’s Note

All claims expressed in this article are solely those of the authors and do not necessarily represent those of their affiliated organizations, or those of the publisher, the editors and the reviewers. Any product that may be evaluated in this article, or claim that may be made by its manufacturer, is not guaranteed or endorsed by the publisher.
